# Bazedoxifene as a Potential Cancer Therapeutic Agent Targeting IL-6/GP130 Signaling

**DOI:** 10.3390/curroncol31100426

**Published:** 2024-09-25

**Authors:** Changyou Shi, Taylor Bopp, Hui-Wen Lo, Katherine Tkaczuk, Jiayuh Lin

**Affiliations:** 1 Department of Biochemistry and Molecular Biology, School of Medicine, University of Maryland, Baltimore, MD 21201, USA; changyou.shi@som.umaryland.edu (C.S.); taylor.bopp@som.umaryland.edu (T.B.); 2 Department of Neurosurgery, McGovern Medical School, University of Texas Health, Houston, TX 77030, USA; hui-wen.lo@uth.tmc.edu; 3 Marlene and Stewart Greenebaum Cancer Center, School of Medicine, University of Maryland, Baltimore, MD 21201, USA; ktkaczuk@umm.edu

**Keywords:** Bazedoxifene, GP130, IL-6, STAT3, cancer therapy

## Abstract

Targeting the interleukin-6 (IL-6)/glycoprotein 130 (GP130) signaling pathway holds significant promise for cancer therapy given its essential role in the survival and progression of various cancer types. We have identified that bazedoxifene (BZA), a Food and Drug Administration (FDA)-approved drug used for the prevention of postmenopausal osteoporosis, when combined with conjugated estrogens in Duavee, also has a novel function as an inhibitor of IL-6/GP130 interaction. BZA is currently under investigation for its potential anticancer therapeutic function through the inhibition of the IL-6/GP130 pathway. Numerous studies have highlighted the efficacy of BZA (monotherapy or combined with other chemotherapy drugs) in impeding progression across multiple cancers. In this review, we mainly focus on the anticancer activity of BZA and the underlying anticancer mechanism through inhibition of the IL-6/GP130 pathway, aiming to provide valuable insights for the design and execution of further research and the potential repositioning of BZA in oncological clinical trials.

## 1. Introduction

The interleukin-6 (IL-6) family of cytokines comprises a group of structurally related proteins that play diverse roles in immune regulation, inflammation, and tissue homeostasis [1]. As a key mediator of inflammation and immunity, IL-6 binds to its receptor IL-6Rα, forming a complex that further engages glycoprotein 130 (GP130), leading to the activation of downstream signaling cascades, including the Janus kinase/signal transducer and activator of transcription (JAK/STAT), Ras/Raf/mitogen-activated protein kinase (MAPK), and phosphatidylinositol 3-kinase (PI3K)/AKT (also known as protein kinase B or PKB) pathways [2]. These intracellular signaling pathways regulate the gene expression patterns involved in cell proliferation, survival, angiogenesis, and metastasis in cancer [3]. Aberrant activation of the IL-6/GP130 signaling pathway is commonly observed in various cancer types, contributing to tumor growth and resistance to therapy [4].

Given its central role in cancer biology, targeting the IL-6/GP130 pathway has emerged as a promising therapeutic strategy for cancer treatment, with ongoing research focusing on developing novel inhibitors to effectively disrupt aberrant signaling and improve patient outcomes.

Several agents targeting the IL-6/GP130 pathway have been developed and investigated for cancer treatment. These include monoclonal antibodies against IL-6 or its receptor (IL-6R), such as siltuximab [5] and tocilizumab [6], which block IL-6 signaling by preventing its interaction with the IL-6 receptor. Nevertheless, they do not impact the dimerization of GP130 molecules, as the signaling of the IL-6/IL-6R axis is fully inhibited upon repression of this dimerization step. Kim et al. (2015) found that tocilizumab can activate the NFκB pathway in non-small cell lung cancer cells, which is contrary to the therapeutic objectives [7]. Additionally, small molecule inhibitors targeting the GP130 receptor, such as raloxifene and bazedoxifene (BZA), have shown promising anticancer effects by inhibiting downstream signaling pathways activated by IL-6 [8,9,10]. Other approaches, including inhibitors of Janus kinases (JAKs) and downstream effectors like STAT3 have shown potential in preclinical and clinical studies for the treatment of various cancers [11,12].

BZA is an estrogen receptor (ER) ligand, and its synthesis involves utilizing raloxifene (RAL) as a structural template and replacing the benzothiophene core with an indole ring, resulting in a compound with unique properties [13]. BZA, a third-generation selective estrogen receptor modulator (SERM), has been approved by the FDA for the prevention and treatment of postmenopausal osteoporosis [14]. In a study by Lewis-Wambi et al. (2011), BZA combined with conjugated estrogen was shown to reduce the proliferation of estrogen receptor (ER)-positive breast cancer cells by downregulating ERα and cyclin D1 [15]. BZA was also identified as a novel inhibitor of IL-6 and GP130 protein-protein interactions through multiple ligand simultaneous docking and drug repositioning approaches [8]. Numerous studies have highlighted the efficacy of BZA through the IL-6/GP130 pathway in breast cancer and many other cancer types. BZA can also enhance the efficacy of other anticancer treatments, including chemotherapy and targeted therapies. It can work synergistically to improve the overall therapeutic outcomes. In this review, we mainly focus on the anticancer activity of BZA and its mechanism of action through the inhibition of the IL-6/GP130 pathway, with the aim of providing valuable insights for the design and execution of further research and the potential repositioning of BZA in oncological clinical trials.

## 2. IL-6/GP130 Signaling Pathway in Cancer

The IL-6/GP130 signaling pathway is increasingly being recognized as a key driver in the development, survival, and drug resistance of various cancers, making it a promising target for cancer therapy. IL-6 plays diverse roles in inflammatory and immune responses as well as in promoting cell survival and proliferation in both normal and tumor cells [16,17,18]. Growing evidence suggests that elevated IL-6 levels are strongly correlated with poor prognosis, reduced survival, and increased metastasis in cancer patients [16,19].

**Figure 1:**
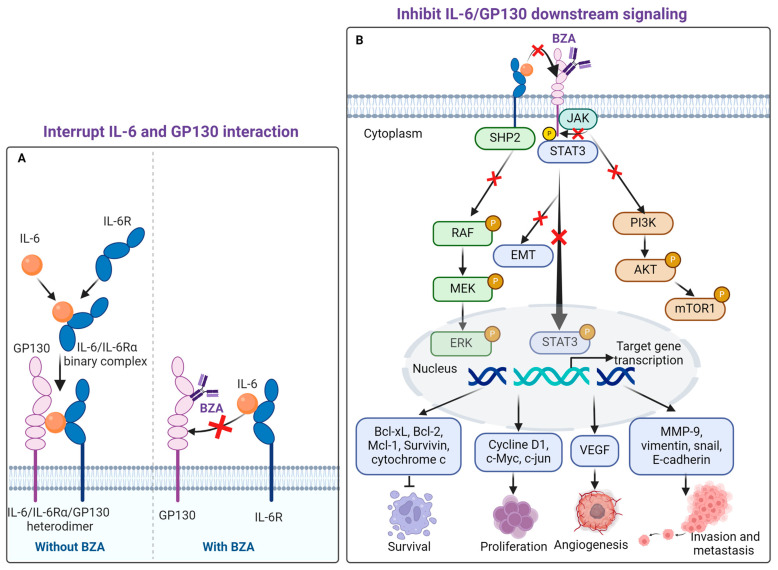
BZA interrupts IL-6 and GP130 interaction and inhibits IL-6/GP130 downstream signaling (**A**) IL-6 binds with IL-6R to create a binary complex, the interaction between GP130 and the IL-6–IL-6 receptor-α (IL-6Rα) binary complex forms the IL-6/IL-6Rα/GP130 heterodimer (left panel, on the left side of the dashed line). By binding to GP130 directly, BZA prevents the interaction of IL-6 with GP130. This inhibition blocks the formation of the IL-6/GP130 receptor complex (right panel, on the right side of the dashed line), and (**B**) IL-6 binds to GP130, leading to the activation of several cell signaling pathways, including JAK/STAT3, PI3K/AKT, and Ras/Raf/MAPK. BZA inhibits STAT3 signaling by either blocking the IL-6/GP130 interaction or preventing the phosphorylation of JAK. Additionally, BZA reduces AKT and MAPK signaling by decreasing the phosphorylation of AKT and ERK, respectively. These pathways are crucial for transcription of genes associated with cell survival, proliferation, angiogenesis, invasion, and metastasis.

GP130 is a transmembrane protein that functions as both a receptor subunit and a receptor-associated signal transducer, shared by the IL-6 family of cytokines. As a signal transducer, GP130 plays a crucial role in uncontrolled proliferation, resistance to apoptosis, and metastasis of various cancers [20,21,22]. When IL-6 binds to its receptor IL-6R, it forms a binary complex that interacts with GP130, resulting in the formation of the IL-6/IL-6Rα/GP130 heterodimer (Figure 1A). This complex initiates several downstream signaling cascades, including the JAK/STAT3, Ras/Raf/MEK/ERK, and PI3K/AKT pathways [23,24,25]. STAT3 is a key downstream regulator of IL-6 signaling and plays a pivotal role in controlling inflammation and promoting neoplastic transformation. Co-overexpression of IL-6 and phosphorylated STAT3 (pSTAT3) is commonly observed in breast cancer and other cancer types [26,27,28].

When the IL-6/IL-6Rα/GP130 complex is established, a Janus kinase (JAK1-JAK3) family of tyrosine kinases is recruited to the membrane, initiating phosphorylation of both the cytoplasmic tail of GP130 and STAT3 [29]. Upon phosphorylation, activated STAT3 undergoes a conformational change, separates itself from the receptor complex, and forms homodimers. This process allows STAT3 to translocate to the nucleus, where it binds to DNA [30] and activates the transcription of oncogenes such as cyclin D1, Bcl-2, Bcl-xL, VEGF, VEGFR2, and matrix metalloproteinases (MMPs) [31,32,33]. These genes are involved in cancer initiation, cell survival, proliferation, angiogenesis, and resistance to apoptosis triggered by conventional therapies [34,35,36,37]. Persistent phosphorylation of STAT3 has been commonly observed in various human cancers and plays a crucial role in supporting tumor survival and growth [38,39,40].

## 3. Bazedoxifene Is a Novel Inhibitor of IL-6/GP130 Signing

Bazedoxifene (BZA), recognized as a selective estrogen receptor alpha (ER-α) modulator, received approval from the U.S. Food and Drug Administration (FDA) in 2013. It was sanctioned for use as part of a combination drug therapy designed to alleviate vasomotor symptoms, such as hot flashes and night sweats, which are commonly associated with menopause [41]. Additionally, as a combination drug, BZA is employed in the prevention of osteoporosis in postmenopausal women, helping to maintain bone density and reduce the risk of fractures [42].

Bazedoxifene (BZA), a third-generation estrogen receptor modulator, offers greater selectivity and an improved safety profile compared to tamoxifen, which is linked to a heightened risk of uterine cancer as a major side effect [13,43]. Phase III clinical trials have shown that BZA provides a favorable reproductive safety profile, with no adverse effects on the endometrium, ovaries, or breasts in postmenopausal women, even with long-term use over three to seven years [44,45].

Through a novel drug discovery approach that integrates Multi-Level Screening and Docking (MLSD) and drug repositioning to target GP130, Li et al. (2014) repurposed BZA as an FDA-approved drug with a unique function—the inhibition of IL-6 and GP130 protein-protein interactions (Figure 1A). The Drug Affinity Responsive Target Stability (DARTS) assay validated the direct binding of bazedoxifene (BZA) to GP130. Additionally, docking studies have shown that BZA’s indole moiety and its seven-membered azepanyl ring effectively compete with IL-6 for binding to GP130 [8].

**Figure 2:**
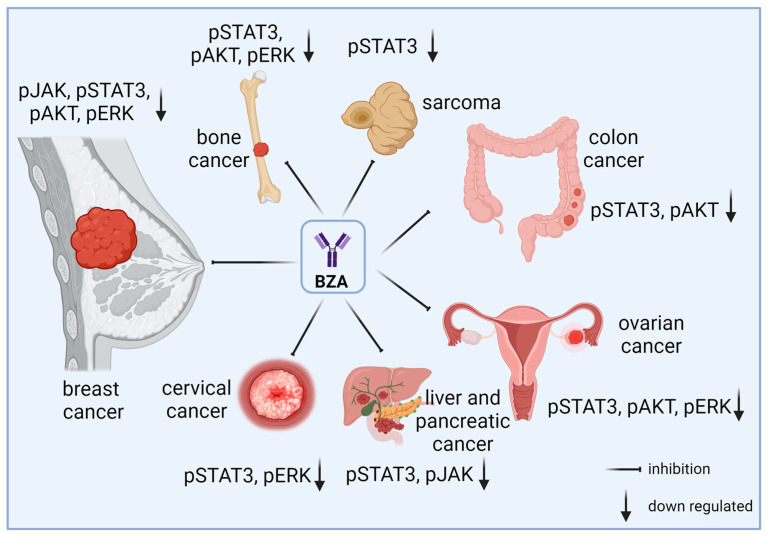
Main downstream effectors of IL-6/GP130 targeted by BZA in multiple human cancers. By disrupting the IL-6/GP130 interaction, multiple downstream regulators are affected, leading to the inhibition of tumor progression in breast cancer and many other cancer types.

Since 2005, the potential anticancer activity of BZA has been explored in various studies (Figure 2). By modulating estrogen receptor activity, BZA inhibits the growth of estrogen-sensitive cancer cells, such as breast cancer [10,46,47]. Many studies have suggested that BZA may inhibit the activation of STAT3 by blocking IL-6 and GP130 interactions [48,49]. BZA may also inhibit additional intracellular pathways linked to cell survival and proliferation (Figure 1B), including Ras/Raf/MEK/ERK and PI3K/AKT. The PI3K pathway is critical for cell growth and survival, and it is often activated by growth factors and hormones like estrogen [50]. Bazedoxifene’s modulation of estrogen receptors can decrease the activation of PI3K, leading to lower levels of phosphorylated AKT, which is a key downstream target. This reduces cell survival signals and promotes apoptosis in breast cancer and other cancers [3]. The Ras/MAPK pathway is activated by the binding of growth factors to receptors like EGFR. Bazedoxifene’s action on estrogen receptors can interfere with this signaling, reducing the activation of Ras. ERK, a key component of the Ras/MAPK pathway, regulates gene expression by phosphorylating various transcription factors, including c-Jun, c-Myc, and CREB [51]. By reducing ERK activation, bazedoxifene can inhibit the transcription of genes involved in cell cycle progression, thereby reducing cancer cell proliferation [52].

The invasion and metastasis of epithelial malignancies are linked to the acquisition of characteristics associated with epithelial-mesenchymal transition (EMT) [53]. As a sub-signal of STAT3 [54], BZA also affects EMT signaling in the brain [55] and in cervical cancer [56]. Modulating these pathways can contribute to G1 cell cycle growth arrest and apoptosis (programmed cell death) by inducing caspase-3 and caspase-7 cleavage in many cancer types, including breast cancer [47], gastrointestinal cancer [52], cervical cancer [56], colon cancer [57], pancreatic cancer [58], hepatocellular carcinoma [59], ovarian cancer [60], lung cancer [61]. Additionally, in rhabdomyosarcoma cancer, BZA has been found to inhibit angiogenesis [62], the process of new blood vessel formation that tumors need for growth and metastasis, thereby slowing tumor progression.

## 4. Effect of Bazedoxifene on Breast Cancer

Breast cancer is the most commonly diagnosed cancer among women worldwide, with millions of new cases each year [63]. In 2023, the American Cancer Society reported approximately 297,790 new cases of invasive breast cancer in women and over 2800 cases in men, resulting in 43,170 deaths in women and 530 deaths in men. Breast cancer is categorized into five major molecular subtypes (Table 1) based on the expression of three hormone receptors: progesterone (PR), estrogen (ER), and human epidermal growth factor receptor 2 (HER2) [64,65].

Comprising 80% of all breast cancer subtypes, luminal tumors are characterized by hormone receptor positivity. These tumors may benefit from hormonal therapy and chemotherapy [66,67]. The HER2-enriched subtype is characterized by greater aggressiveness and rapid growth. It has a worse prognosis compared to luminal breast tumors and requires specific drugs targeting the HER2/neu protein, such as trastuzumab, pertuzumab, and tyrosine kinase inhibitors [68]. Triple-negative breast cancer (TNBC) is defined by the absence of ER, PR, and HER2 expression. This subtype is known for its aggressive nature, rapid metastasis, and the poorest five-year survival rate among patients with breast cancer [69]. Traditional hormone therapies and HER2-targeted treatments are ineffective against TNBC due to its lack of ER, PR, and HER2, leaving patients with limited treatment options [70].

Table 2 summarizes the antitumor effects of BZA on breast cancer, including its biological effects and molecular targets. As a selective estrogen receptor degrader in hormone replacement therapies, BZA demonstrates enhanced inhibitory potency against ERα mutants (Y537S and D538G) compared to tamoxifen and exhibits additional inhibitory activity in ER+ breast cancer cell lines when combined with the CDK4/6 inhibitor palbociclib [46] by inducing a G1 blockade in hormone-independent MCF-7:5C cells. Bazedoxifene (BZA) uniquely inhibits the growth of hormone-independent MCF-7:5C cells breast cancer cells by downregulating key proteins such as ERα and cyclin D1; this action distinguishes BZA from other selective estrogen receptor modulators, including raloxifene, 4-hydroxytamoxifen (4OHT), endoxifen (ENDOX), and fulvestrant [15]. Fu et al. (2019) explored the inhibitory effects of BZA, both alone and in combination with paclitaxel, a first-line treatment for breast cancer, in various ER-positive breast cancer cell lines (MCF7, T47D, and BT474) and TNBCs (MDA-MB-231, MDA-MB-468, and 4T1). Their study revealed that BZA effectively reduced the viability of both ER+ and TNBC cells. Moreover, when combined with paclitaxel, BZA demonstrated enhanced inhibition of cell viability, colony formation, and cell migration in vitro, as well as more pronounced suppression of tumor growth in TNBC models in vivo, compared to either drug used alone [47]. Another study demonstrated similar findings, showing that BZA alone inhibited the viability, survival, proliferation, and migration of triple-negative breast cancer (TNBC) cells. This inhibition was achieved through decreased phosphorylation of STAT3 and the downstream targets of the IL6-GP130 pathway, including AKT and ERK. Additionally, oral administration of BZA significantly suppressed tumor growth in TNBC xenograft mice [10].

## 5. Effect of Bazedoxifene in Other Types of Cancer

### 5.1. Colon Cancer

Colon cancer is notable as the third most common malignant tumor worldwide [71]. According to the 2022 report from the International Agency for Research on Cancer (IARC), there were more than 1.9 million new cases of colorectal cancer (CRC) and over 900,000 deaths globally. CRC constituted 10.0% of the overall cancer incidence [72]. Chemotherapy is the classical and most commonly used treatment for patients with CRC. Nevertheless, the development of drug resistance is a common occurrence in most patients with CRC [73]. Li et al. (2018) demonstrated that bazedoxifene (BZA) significantly enhances the antitumor effects of 5-fluorouracil (5-FU), a commonly used chemotherapeutic agent in colon cancer, by inhibiting the IL-6/GP130 signaling pathway and reducing the phosphorylation of STAT3, AKT, and ERK in colon cancer cells. Consistent with the in vitro results, the combination of 5-FU and BZA exhibited a synergistic antitumor effect in a colon cancer xenograft mouse model [74]. Additionally, Wei et al. (2019) evaluated BZA’s efficacy in colon cancer cells and found that BZA, either alone or in combination with oxaliplatin (L-OHP), significantly induces apoptosis, inhibits cell viability and migration, and reduces tumor burden in HCT-15 and DLD-1 xenograft models (Table 3). They also observed that BZA inhibits the phosphorylation of STAT3 and the nuclear translocation of pSTAT3 induced by IL-11 in colon cancer cells [57].

### 5.2. Ovarian Cancer

Ovarian cancer has the highest mortality rate among gynecological cancers and is the fifth most common cause of cancer-related deaths among women in the United States [77]. In 2023, the American Cancer Society reported 19,710 new cases of ovarian cancer in the United States, with the disease claiming the lives of 13,270 women. The main treatment for ovarian cancer typically involves a combination of radical surgery and adjuvant chemotherapy [78]. Park et al. (2022) found that combining bazedoxifene (BZA) with the chemotherapy agent paclitaxel significantly reduced cell viability, migration, and invasion in ovarian cancer cells. This combination also demonstrated the ability to inhibit human ovarian cancer growth in a xenograft tumor model. Additionally, the treatment was shown to suppress IL-6-mediated GP130/STAT3 signaling, induce apoptosis in ovarian cancer cells, and inhibit the EMT process [60]. Poly (ADP-ribose) polymerase (PARP) inhibitors have emerged as a significant class of drugs for the treatment of ovarian cancer with BRCA mutations by preventing the repair of single-stranded DNA breaks. However, most ovarian cancer patients do not have BRCA mutations, which limits the use of PARP inhibitors [79]. Zhang et al. (2021) demonstrated that the combination of BZA with talazoparib, a PARP inhibitor, synergistically inhibited cell viability, migration, and colony formation in ovarian cancer cells independent of BRCA mutation status. This drug combination also led to the suppression of p-AKT, c-Myc, p-ERK, and ERα expression while inducing the expression of γH2AX, a biomarker indicative of DNA double-strand breaks [75].

### 5.3. Other Cancer Types

Bazedoxifene (BZA) exhibits promising antitumor effects in various types of cancer beyond breast cancer, colon cancer, and ovarian cancer (Table 3). Xu et al. (2016) found that resistance to common chemotherapeutic agents, such as doxorubicin, cisplatin, and MEK inhibitors, was observed in rhabdomyosarcoma cells with elevated levels of phosphorylated STAT3. Inhibiting GP130/STAT3 signaling with BZA can sensitize these cancer cells to treatment, thereby reducing cell viability and inducing apoptosis [48]. BZA treatment reduced the viability and proliferation of pancreatic cancer cells by interfering with IL-6/GP130 signaling. This blockade disrupts key metabolic processes, including glycolysis, and diminishes the cells’ ability to form colonies, indicating a decrease in their oncogenic potential [49]. BZA was found to inhibit cell viability, wound healing, and colony formation in various liver cancer cell lines (HEPG2, 7721, and HUH-7). Furthermore, the study revealed a significant inhibition of tumor growth in HEPG2 mouse xenografts following daily oral administration of BZA. The underlying mechanism involved the reduction of IL-6-induced STAT3 phosphorylation by BZA [59]. Similarly, BZA showed activity in brain tumors, such as glioblastoma multiforme (GBM), which is a highly aggressive brain tumor that arises from astrocytes. BZA, either alone or in conjunction with paclitaxel, demonstrates the ability to inhibit cell survival and invasion of glioblastoma cells by inducing apoptosis and mitigating EMT. Moreover, BZA, combined with other chemotherapeutic agents, such as paclitaxel, accelerates the phosphorylation and inactivation of YAP (Yes-associated protein), a key regulator of cell proliferation and survival [55]. Similarly, BZA can modulate the EMT signaling pathway and reduce cell migration and invasion in HPV-positive cervical cancer cells [56]. Moreover, BZA has shown efficacy against osteosarcoma tumors, where it inhibits cell proliferation, induces cell cycle arrest, and promotes apoptosis by inhibiting IL-6 and IL-11/GP130 signaling, suggesting its potential as a therapeutic agent for bone malignancies [76]. These findings underscore the broad spectrum of antitumor effects of BZA across different cancer types, highlighting its potential as a promising therapeutic option for diverse malignancies.

## 6. Clinical Trials of BZA in Cancer

Current clinical trials on BZA as an antitumor agent have focused on breast cancer (Table 4). A pilot study in 2019 spanned six months to explore the feasibility of a tissue-selective estrogen complex composed of bazedoxifene (20 mg) and conjugated estrogen (0.45 mg; Duavee) on risk biomarkers for postmenopausal breast cancer in women deemed high-risk [80]. The results of this study revealed a notable decrease in mammographic fibroglandular volume, indicating a reduction in dense breast tissue, which is a known risk factor for breast cancer. Additionally, there was a significant reduction in cellular proliferation within the breast tissue of women who had a baseline Ki-67 index of 1% to 4%, a marker that measures cell growth and replication rates. Furthermore, combination therapy exhibited beneficial effects on several serum biomarkers, such as PR, testosterone, and insulin-like growth factor 1 (IGF-1). These findings indicate that BZA, in combination with conjugated estrogen, may potentially lower the risk of breast cancer in high-risk postmenopausal women [80]. Another recent clinical study aimed to evaluate the safety, tolerability, and efficacy of BZA in combination with palbociclib in patients with advanced hormone receptor-positive breast cancer. The combination therapy was well tolerated and showed promise, with a significant proportion of patients experiencing tumor shrinkage and extended progression-free survival [81]. There are two other clinical trials that are ongoing, which are summarized in Table 4. Given these promising results, it is recommended to conduct further placebo-controlled Phase IIB trials to validate these findings and to better understand the potential of combination therapies for breast cancer and other cancer types.

## 7. Limitation of BZA in Cancer

Bazedoxifene (BZA) has demonstrated effectiveness in preclinical models, particularly in breast cancer, but its efficacy may be limited to hormone receptor-positive cancers. While preclinical studies and early phase clinical trials have shown promise, there are still limited clinical data on the efficacy and safety of bazedoxifene as an anticancer agent in cancer types other than breast cancer. Larger and more comprehensive trials are needed to establish its role in cancer therapy. Bazedoxifene (BZA) often needs to be combined with other therapeutic agents, such as CDK4/6 inhibitors (e.g., palbociclib) or other targeted therapies, to achieve significant antitumor effects. This combination approach can complicate treatment regimens and lead to increased side effects and drug interactions. Like other endocrine therapies, the prolonged use of BZA might lead to the development of resistance, which limits its long-term efficacy and necessitates the development of additional treatment strategies. Although BZA is generally well tolerated, its use in cancer therapy may lead to side effects, including those related to its estrogen-modulating activity. These effects can include hot flashes, thromboembolic events, and other hormone-related symptoms, which can impact patient compliance and quality of life.

## 8. Conclusions and Future Perspectives

IL6/GP130 is emerging as a promising therapeutic target in cancer treatment, as increasing evidence highlights its critical role in tumor development, proliferation, metastasis, and chemoresistance in various cancers. Bazedoxifene (BZA), a third-generation SERM, was approved by the FDA in 2007 for the prevention of osteoporosis in postmenopausal women when used in combination with conjugated estrogens in Duavee. It is currently being investigated for its anticancer properties, both in vivo and in vitro, across multiple cancer types. Several observations underscore BZA’s potential of BZA as a leading anticancer agent: (1) given its strong modulation of estrogen receptors, BZA holds promise for breast cancer and ovarian cancer treatment; (2) it exhibits broad spectrum cytotoxicity against multiple cancer types by blocking the cell cycle at the G1 phase and inducing apoptosis; (3) it modulates several signaling pathways such as IL-6/GP130, JAK/STAT3, and AKT/mTOR, commonly dysregulated in cancer cells, to impede cancer progression; and (4) it increases chemo-sensitizing activity when combined with conjugated estrogen or other chemotherapeutic drugs. However, most studies have only been conducted in cancer cells or preclinical mouse models; therefore, additional efforts are required to evaluate its antitumor effects in clinical studies.

Looking ahead, BZA has considerable promise as a therapeutic agent for cancer treatment. Future research should aim to evaluate its potential as a chemopreventive agent, explore its synergistic effects with other anticancer therapies, and optimize dosing regimens. Additionally, investigating BZA’s role in combination therapies and its ability to overcome drug resistance mechanisms would be highly valuable. Ongoing clinical trials will also provide critical insights into its efficacy, safety profile, and tolerability in patients with cancer, paving the way for its future application in clinical practice.

## Figures and Tables

**Table 1:** Characteristics of breast cancer subtypes.

	Receptor Expression	Frequency, %	5-Year Survival, %	Therapy
Luminal A	ER^+^, PR^+^, HER2^−^	50	94.8	Hormonal
Luminal B	ER^+^, PR^+/−^, HER2^−^	15		Hormonal/Chemo
Luminal/HER	HR^+^, HER2^+^	15	91.0	Hormonal/Chemo
HER enriched	HR^−^, HER2^+^	5	85.6	Hormonal/Chemo
TNBC	ER^−^, PR^−^, HER2^−^	15	77.6	Chemo/Experimental

**Table 2:** BZA treatment for breast cancer.

Cell Lines	Biological Effects	Molecular Target Inhibited	Reference
TNBC: MDA-MB-231, MDA-MB-468, SUM159	(1) BZA alone inhibits cell viability, survival, proliferation, and cell migration in all TNBC cells. (2) Tumor growth in mice was remarkably suppressed by BZA via an oral administration route.	p-STAT3, p-ERT, and p-AKT ↓	[10]
ER+ breast cancer cells: MCF-7, T47D, MCF-7:5C, MCF-7:2A	(1) BZA inhibited the growth of both hormone-dependent and -independent ER-positive breast cancer cells. (2) BZA induced G1 blockade in hormone-independent MCF-7:5C cells.	ERα, cyclin D1 ↓	[15]
ER+ breast cancer cells: MCF-7, ZR75-1 T47D	(1) BZA is a potent inhibitor against somatic mutants of ERα (Y537S and D538G) in breast cancer cells. (2) BZA has additional inhibitory activity in combination with the CDK4/6 inhibitor palbociclib.	ERα, cyclin D1, c-myc, and PR ↓	[46]
ER+ breast cancer cells: MCF7, T47D, BT474. TNBCs: MDA-MB-231, MDA-MB-468, 4T1	(1) BZA inhibited cell viability, clonal formation, migration, and induces apoptosis in both ER+ and TNBC cell lines. (2) BZA alone suppressed tumor growth in the TNBC xenograft model (3) BZA and paclitaxel combination exhibits more potent inhibition of the malignant features of breast cancer than either drug alone.	ER+ breast cancer cells: ERα ↓ TNBC: p-STAT3, p-ERT, and p-AKT ↓	[47]

**Table 3:** BZA treatment for other types of cancer.

Cancer Type and Reference	Cell Lines	Biological Effects	Molecular Target Inhibited
Rhabdomyosarcoma [48]	RH4, RH5, and RH30	(1) BZA inhibited cell migration and induced apoptosis in rhabdomyosarcoma cells. (2) BZA enhanced the sensitivity of rhabdomyosarcoma cells to anticancer drugs such as doxorubicin, cisplatin, or AZD6244 by inhibiting GP130 signaling.	p-STAT3, cytochrome c ↓
Pancreatic cancer [49]	PANC-1, HPAF-II, Capan-1, BxPC-3, and MIA PaCa-2	(1) BZA impeded IL-6 mediated cell viability, proliferation, glycolysis, and colony formation in pancreatic cancer cells.	IL-6R, p-STAT3, AKT1 ↓
Brain cancer [55]	Glioblastoma cells	(1) BZA inhibited glioblastoma cell viability in a dose-dependent manner. (2) When combined with paclitaxel, BZA more effectively suppressed glioblastoma progression by enhancing apoptosis and reducing EMT.	ERα, Cyclin D1, Bcl-2, p-p70S6K, vimentin, MMP9 and snail ↓ Cleaved caspase-3 ↑
Cervical Cancer [56]	HPV-positive cervical cancer cell lines: SiHa, HeLa and CaSki	(1) BZA reduced cell proliferation, colony formation, migration, and invasion while promoting apoptosis in HPV-positive cervical cancer cells. (2) BZA inhibited tumor growth in a dose-dependent manner in the SiHa mouse xenograft model and suppressed the progression of epithelial-mesenchymal transition (EMT).	Bcl-xL, Mcl-1, pGP130, pSTAT3 pERK1/2, β-catenin, vimentin, Wnt5β ↓ Bim, Bax, E-cadherin ↑
Colon cancer [57]	DLD-1, HCT-15, and HCT-116	(1) BZA alone or combined with oxaliplatin can induce apoptosis and inhibit cell viability, cell colony formation, and cell migration in colon cancer cells. (2) BZA (10 mg/kg) alone attenuated HCT-15 xenograft tumor burden.	p-STAT3, p-AKT, Cyclin D1, survivin, c-myc ↓
Hepatocellular carcinoma [59]	Human liver cancer cell lines: Hep3B, HEPG2, SSMC 7721, HUH-7	(1) BZA inhibited cell viability, wound healing, and colony formation while inducing apoptosis in liver cancer cells. (2) In a HEPG2 mouse xenograft model, BZA effectively suppressed tumor growth.	p-STAT3, p-JAK1, p-JAK2, Bcl-2, surviving ↓ Translocation of STAT3 ↓ Cleaved caspase-3 ↑
Ovarian cancer [75]	SKOV3, UWB1.289 (BRCA1-null) and OV75	(1) BZA combined with PARP inhibitor talazoparib synergistically inhibits cell viability, cell migration, cell growth, and cell colony formation on all ovarian cell lines.	p-AKT, c-myc, p-ERK, ERα ↓ γ-H2AX ↑
Bone tumor [76]	Osteosarcoma cancer cell lines: SJSA, SaoS2, 143B	(1) BZA reduced cell viability and migration in osteosarcoma cells by inhibiting IL-6 and IL-11/GP130 signaling pathways. (2) When combined with temsirolimus, BZA synergistically suppressed osteosarcoma progression in both in vitro and in vivo models.	p-STAT3, p-ERK1/2, p-AKT, survivin ↓ Cleaved caspase-9 ↑

**Table 4:** Completed and ongoing clinical trials of BZA as an antitumor agent.

Title	Phase	Intervention	Cancer Type	Outcome
Bazedoxifene and conjugated estrogens for the prevention of breast cancer in peri- or postmenopausal women at increased risk for development of breast cancer	Phase II	BZA + conjugated estrogens	Breast Cancer Prevention	1. a significant reduction in mammographic fibroglandular volume, serum progesterone, bioavailable testosterone, and IGF-1 levels, as well as an increase in bioavailable estradiol. 2. a reduction in Ki-67 levels for women in the higher-risk cohort 3. an improvement in hot flash symptoms and overall menopause-related quality of life.
A phase Ib/II study of palbociclib in combination with bazedoxifene in hormone Receptor-positive breast cancer	Phase Ib/II	Palbociclib + BZA	Hormone Receptor-Positive (HR+) Breast Cancer	1. Promising clinical efficacy in patients with advanced HR+ breast cancer, with some patients experiencing tumor shrinkage or disease stabilization. 2. The treatment regimen was generally well tolerated, with manageable side effects consistent with those typically seen in palbociclib therapy, such as neutropenia, fatigue, and gastrointestinal symptoms.
A large-scale multicenter Phase II study evaluating the protective effect of a tissue-selective estrogen complex (TSEC) in women with newly diagnosed ductal carcinoma in situ	Phase II	BZA + conjugated estrogens	Ductal Carcinoma in Situ (non-invasive or pre-invasive breast cancer)	Ongoing, 1. investigated the proliferation of cancer cells (Ki-67) and expression of hormone receptors such as ERα, PR, and HER-2, along with other markers related to tumor progression. 2. examined the impact of CE/BZA on quality of life, assessing potential side effects and menopausal symptom relief.
Bazedoxifene as a concomitant treatment of patients with metastatic pancreatic adenocarcinoma	Phase II	BZA + gemcitabine with or without nab-paclitaxel	Metastatic pancreatic adenocarcinoma with IL6/GP130/STAT3 pathway activity	Ongoing, 1. measures changes in the expression of the IL-6/GP130/STAT3 pathway and tumor markers (such as CA 19-9) 2. evaluate patient quality of life. 3. Toxicity and safety assessments.
